# Growth promotion of *Euglena gracilis* by ferulic acid from rice bran

**DOI:** 10.1186/s13568-018-0547-x

**Published:** 2018-02-08

**Authors:** Jiangyu Zhu, Minato Wakisaka

**Affiliations:** 1 Graduate School of Life Science and Systems Engineering, Kyushu Institute of Technology, 2-4 Hibikino, Fukuoka, 808-0196 Japan; 2 School of Food Science and Engineering, Yangzhou University, No. 196 Huayang West Road, Hanjiang District, Yangzhou, 225127 Jiangsu China

**Keywords:** *Euglena gracilis*, Ferulic acid, Photosynthetic pigments, Paramylon

## Abstract

A significant growth promotion of *Euglena gracilis* was achieved by simply adding ferulic acid from rice bran without diminishing the accumulation of valuable products like paramylon. *E. gracilis* is a freshwater microalga that is widely applied in cosmetics, food, medicine, and supplements, and it is considered a potential source of biofuel. It is therefore important to enhance its yield at a lower cost for its commercial viability. Introducing a growth regulator derived from agro waste is considered a cheaper and safer strategy to improve biomass productivity compared with other alternatives such as implementing genetic engineering or adding nutrients and plant hormones as growth stimulator. The effect of ferulic acid derived from rice bran on the growth and metabolism of *E. gracilis* was investigated in this study. To aid in the dissolution of ferulic acid, 1% dimethyl sulfoxide (DMSO) was added to Cramer–Myers medium. Ferulic acid could alleviate the inhibitory effect of DMSO and significantly promoted the growth of *E. gracilis*. It was found that cell density was 2.5 times greater than that of the control group and 3.6 times greater than that of the negative control group when 500 mg/L of ferulic acid was added. In addition, the photosynthetic pigment content, especially chlorophyll a, increased with increasing ferulic acid concentrations. The total paramylon production would also be enhanced by ferulic acid since the number of cells increased without reducing the cellular content of paramylon.

## Introduction

*Euglena gracilis* is a common primary producer in aquatic ecosystems where it produces a large biomass with beneficial metabolites that have a high commercial value. Therefore, it has been widely applied in many fields, such as in food, supplements, and cosmetics (Gong et al. 2011; Sanghvi and Martin 2010). *E. gracilis* can produce a unique metabolite called paramylon (β-1,3 glucan), which is a glucose polymer with β-1,3 linkages structurally similar to starch. Recently, its medical potential has been reported, for instance, paramylon and its chemically modified derivatives exhibit a certain degree of anti-HIV activity (Sakagami et al. 2012), antitumor activity (Xiao et al. 2004), and anti-infection activity (Chan et al. 2009). Therefore, the market demand for *E. gracilis* is expected to increase in the future.

However, under a natural culture, growth speed and metabolite accumulation of microalgae are very low and strictly limited by the culture conditions, such as light (Ogbonna et al. 1995), pH (Danilov and Ekelund 2001), and temperature (Kitaya et al. 2005). Researchers have spent a significant amount of time and effort to overcome these problems. From a micro perspective, gene editing seems to be a good solution and this topic is now very popular. Theoretically, by modifying some functional genes, we can stably enhance yield and even improve the properties against bacteria or diseases (Gan and Maggs 2017). From a practical perspective, however, the cost of this method is extremely high and the process is more complex than normal approaches. More importantly, the safety of the genetically modified microorganisms is still controversial and the method should be severely restricted (Araki et al. 2014). From a macro point of view, the most common method used to improve growth and metabolism is supplementing with various types of additives, for example, exogenous nutrients (Ogbonna et al. 1998) and plant hormones (Noble et al. 2014). Nevertheless, these additives are still costly. It was reported that 1000 mg/L of alginate oligosaccharides derived from macroalgae could promote the growth of *Spirulina platensis* 3.68-fold (Nogami et al. 2017), and 5000 mg/L of steel-making slag, a byproduct of steel processing, could increase the growth of *S. platensis* 1.12-fold (Nogami et al. 2016). These studies suggested a new and promising strategy to enhance microalgae yield at a low cost with high efficiency by simply adding unutilized or waste materials. In consideration of a safe environmental application and cost, we selected ferulic acid from rice bran as a potential growth promoter. Ferulic acid is ubiquitous in plant cell wall components and is present in a wide range of natural sources. With the discovery of more physiological functions of ferulic acid, including antioxidant (Maurya and Devasagayam 2010), anti-inflammatory (Roy et al. 2013), and antitumor activities (Yang et al. 2015), it is becoming more widely used in medicine, healthcare, and cosmetics. Similar to other phenols, ferulic acid is an antioxidant that can scavenge reactive oxygen species (ROS). Free radicals and ROS are known to be involved in DNA damage and cell aging, resulting in unavoidable growth issues (Cooke 2003; Mallick and Mohn 2000). Ferulic acid was expected to alleviate oxidative stress and provide suitable growth conditions for *E. gracilis* cells.

The aim of this study was to investigate the effect of ferulic acid from rice bran on *E. gracilis* growth, morphology, and metabolism. This was achieved by analyzing *E. gracilis* physiological parameters, such as cell density, dry weight, cell length, pigments, and paramylon content.

## Materials and methods

### Culture conditions

*Euglena gracilis* Klebs strain (NIES-48) purchased from National Institute for Environmental Studies of Japan was cultured in Cramer–Myers (CM) medium with the following composition (mg/L): (NH_4_)_2_HPO_4_, 1000; KH_2_PO_4_, 1000; MgSO_4_·7H_2_O, 200; CaCl_2_·2H_2_O, 20; FeSO_4_·7H_2_O, 3; MnCl_2_·4H_2_O, 1.8; CoSO_4_·7H_2_O, 1.5; ZnSO_4_·7H_2_O, 0.4; Na_2_MoO_4_·2H_2_O, 0.2; CuSO_4_·5H_2_O, 0.02; Vitamin B_12_, 0.0005; Thiamine HCl, 0.1. Ferulic acid extracted from rice bran was obtained from Tsuno Rice Fine Chemicals Co., Ltd. (Wakayama, Japan). Dimethyl Sulfoxide (DMSO) was used as an auxiliary solvent to improve the solubility and bioavailability of ferulic acid. For cell culture, 10 mL *E. gracilis* cells in the exponential phase were inoculated into Erlenmeyer flasks containing 100 mL CM medium. Different concentrations of ferulic acid dissolved in DMSO stock solution were added. Finally, the DMSO concentration of experimental groups was adjusted to 1%. A negative control group was additionally set to evaluate the effect of the auxiliary solvent DMSO. All groups were cultured at 25 ± 1 °C under a 5000 lx light intensity (12:12 h light–dark cycle). Throughout the cultivation, flasks were shaken evenly by hand three times per day to avoid cell aggregation. In addition, the pH of the medium was determined periodically by pH meter (LAQUA-2103AL, Horiba, Japan).

### Cell growth

Cell density and dry weight were determined to assess growth. Cell counting was carried out periodically using a hemocytometer chamber. The dry weight was measured by the following method. After completion of the culture, the algal cells were harvested by centrifugation at 10,000 rpm for 15 min and then washed twice with distilled water. Cells were deposited on filter paper (GC-50, Advantec, Japan) after filtration, dried at 120 °C for 1 h, and transferred to a desiccator for cooling to room temperature. Dry weight was calculated by comparing the difference between before and after drying.

### Cell morphology

Cell morphology of *E. gracilis* was observed and recorded by microscope (Model BA210a, Motic, Japan). Cell lengths of more than 200 cells were measured using image processing software (Motic Image Plus 2.2S) at hours 6 and 12 of the light and dark cycle, respectively.

### Metabolites assay

Chlorophyll a, chlorophyll b and carotenoid content was estimated according to Lichtenthaler’s procedures (Lichtenthaler and Wellburn 1983). Pigments were extracted with 80% (v/v) acetone and the optical density was measured by a spectrophotometer (UV–vis 1200, Shimadzu, Japan) at wavelengths of 470, 645, and 663 nm.

Paramylon was extracted and purified according to Barsanti’s method (Barsanti et al. 2001). For sample preparation, 50 mL aliquots of cultures were centrifuged at 12,000 rpm for 30 min and harvested cells were washed three times with distilled water. Cell pellets were frozen at − 20 °C for 12 h to break the cells, and were then resuspended in a 1% (w/v) sodium dodecyl sulfate and 5% (w/v) Na_2_EDTA solution. The suspension was shaken for a while and incubated at 37 °C for 1 h. Paramylon granules were retrieved by centrifugation for 30 min at 12,000 rpm. The above extraction treatment was repeated until the supernatant was translucent. The obtained paramylon granules were then rinsed three times with 70 °C glass-distilled water and immediately collected on glass fiber filter paper (APFC type, Millipore).

### Statistical analysis

All experiments were performed in triplicate. Data were processed by static processing software SPSS v. 19.0 (Armonk, NY: IBM Corp.) and are presented as the mean ± standard deviation.

## Results

### Growth promotion of *E. gracilis* by ferulic acid

**Fig. 1:**
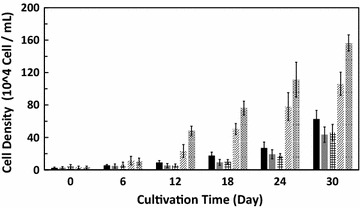
Growth profile of *Euglena gracilis* with different concentrations of ferulic acid. *N.C.* negative control group. Symbols correspond to (Black square) control group, (black square) negative control group, (square with orthogonal crosshatch fill) 10 mg/L ferulic acid, (square with upper right to lower left fill) 100 mg/L ferulic acid, (square with diagonal crosshatch fill) 500 mg/L ferulic acid

This study is the first to report a considerable promotion effect of ferulic acid on microalgal growth. Figure 1 shows the growth profile of *E. gracilis* with various concentrations of ferulic acid; the cell density in the negative control group and 10 mg/L ferulic acid treatment group was significantly lower than that of the control group, indicating that the presence of the auxiliary solvent DMSO had an inhibitory effect on the growth of *E. gracilis*. However, at higher concentrations of ferulic acid, their growth was significantly promoted in a concentration-dependent manner. When the ferulic acid concentration was increased to 100 mg/L, growth was not only restored but was also much higher than that of the control group. At day 30, cell density in the 500 mg/L ferulic acid treatment group showed a 2.5-fold increase compared with that of the control group and a 3.6-fold increase compared to that of the negative control group. The results were statistically significant (p < 0.05).

**Fig. 2:**
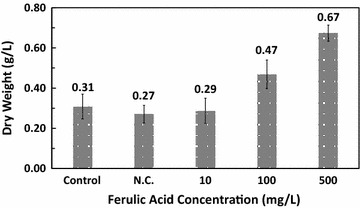
Dry weight of *Euglena gracilis* with different concentrations of ferulic acid. *N.C.* negative control group

The dry weight of biomass from each group was also measured to assess growth (Fig. 2), and the results were consistent with cell density with a slight difference. At a higher concentration of ferulic acid (100 and 500 mg/L), the dry weight (0.47 ± 0.07 g/L and 0.67 ± 0.04 g/L, respectively) were significantly higher than that of the two control groups (0.31 ± 0.06 g/L in the control group and 0.27 ± 0.04 g/L in negative control group). But the difference between the negative control group and the positive control group was not significant in this experiment.

### Effect of ferulic acid on the morphology of *E. gracilis* cells

**Fig. 3:**
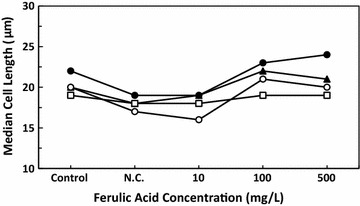
Effect of ferulic acid on the median cell length *Euglena gracilis* under different light conditions. *N.C.* negative control group. Symbols correspond to (black circle) light 6 h, (black up-pointing triangle) light 12 h, (white square) dark 6 h, (white circle) dark 12 h

The variation in median cell length after the addition of ferulic acid is shown in Fig. 3. The median cell length of the negative control group and the 10 mg/L ferulic acid treatment group decreased compared with that of the control group, but at higher concentrations of ferulic acid, cell morphology was restored to normal and the median length was longer than that of the control group. The most noticeable change in cell length was obtained at hour 6 of the light period. Cell length at hour 6 was longer than that of the other three periods, and the median cell length decreased from 22 μm in the control group to 19 μm in the negative control group but increased to 24 μm in the 500 mg/L ferulic acid treatment group.

### Effect of ferulic acid on metabolite content of *E*. *gracilis* cells

**Fig. 4:**
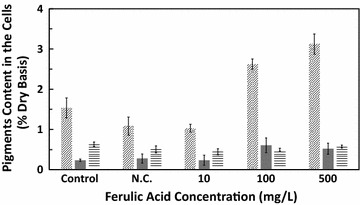
Pigments content in *Euglena gracilis* cells cultured with different concentrations of ferulic acid. *N.C.* negative control group. Symbols correspond to (square with upper right to lower left fill) chlorophyll a, (black square) chlorophyll b, (square with horizontal fill) carotenoid

Dry content of chlorophyll a, chlorophyll b, and carotenoid is shown in Fig. 4. Among the three pigments in the control group, chlorophyll a content was the highest at 1.54 ± 0.25% followed by a carotenoid content of 0.63 ± 0.06%, whereas chlorophyll b content was the lowest, accounting for 0.24 ± 0.03%. The chlorophyll a content in the negative control group was significantly decreased. In the ferulic acid treatment groups, chlorophyll a content was restored and increased in a dose-dependent manner with increasing ferulic acid concentrations, finally reaching 3.12 ± 0.78% in the 500 mg/L ferulic acid treatment group. A similar trend was observed in chlorophyll b. However, there was no significant change in carotenoid content throughout the experiment.

**Fig. 5:**
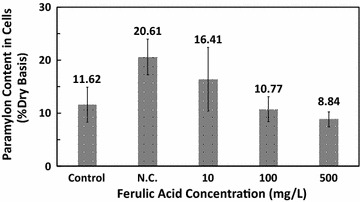
Paramylon content in *Euglena gracilis* cells cultured with different concentrations of ferulic acid. *N.C.* negative control group

The paramylon content of *E*. *gracilis* cells showed opposite trend to chlorophyll content. As shown in Fig. 5, a higher paramylon content of 20.61 ± 3.36% was obtained in the negative control group, whereas in the control group it was only 11.62 ± 3.27%. After the addition of ferulic acid, the paramylon content decreased and eventually reached a normal level. The paramylon content in the 500 mg/L ferulic acid treatment group was comparable to that of the control group, indicating ferulic acid does not diminish the accumulation of paramylon. Total paramylon production would also be enhanced by ferulic acid since the number of cells increased without reducing the cellular content of paramylon.

## Discussion

Here, we are the first to report on a significant growth promotion effect of ferulic acid on *E. gracilis*. As for the particular mechanism, on the one hand, ferulic acid and its oxidative products might serve as an exogenous organic carbon source for *E. gracilis* growth. On the other hand, ferulic acid might play phytohormone-like regulatory roles in cell growth. As we know, *E. gracilis* has characteristics of both animals and plants (Shigeoka et al. 1979). These can grow heterotrophically as well as photosynthesis, and take food from the outside environment via phagocytosis. When nutrient particles reach the cell surface, the cell membrane forms vesicles to entrap the particles for digestion. Ferulic acid might be utilized in this form by cells. Genes responsible for the catabolic mechanism of ferulic acid were identified in *Pseudomonas* sp. as being located on a DNA region with two *Eco*RI fragments, E230 and E94 (Overhage et al. 1999). If the catabolic pathway and related enzymes of ferulic acid were also determined for *E. gracilis*, we could know whether ferulic acid or its oxidative products serve as an exogenous organic carbon source for growth promotion. Further, a phytohormone-like regulation mechanism might also be involved in growth promotion. Whether phytohormones were present in primitive single-cell plants such as microalgae has been controversial, however, the latest study revealed that functional plant hormone metabolic pathways existed in a broad spectrum of microalgal species (Buggeln and Craigie 1971; Lu and Xu 2015). This finding provides a possible explanation for the regulation effect of ferulic acid on *E. gracilis*, since it was reported that ferulic acid could synergize indoleacetic acid (IAA)-induced growth by counteracting IAA decarboxylation (Tomaszewski and Thimann 1966), while IAA has been proved to have a notable promotion effect on different microalgae such as *Anabaena vaginicola*, *Nostoc calcicola,* and *Scenedesmus quadricauda* (Hashtroudi et al. 2012; Kozlova et al. 2017).

The effect of phenolic compounds such as ferulic acid on microalgal growth might be different for algae species. Previously, the inhibitory effect of various phenolic compounds including 11 phenols and acids produced via the shikimate pathway on the blue-green algae *Microcystis aeruginosa* (Nakai 2001) was reported. Compared with other plant-producing phenols and acids which had inhibitory effect, ferulic acid did not affect the growth of *M. aeruginosa*. The inhibitory effect of these phenols depended on their molecular structure, and growth inhibition was mainly attributed to free radicals and ROS produced by the autoxidation of these phenols. As for ferulic acid, the methoxy and phenolic hydroxyl groups on the benzene ring are in the ortho position, which means that the ferulic acid structure is stable and will not be autoxidized easily.

As for the decrease in cell density in the negative control group, DMSO was supposed to have an inhibitory effect on growth. However, the biomass dry weight of the negative control group did not decrease notably, inferring that the production of certain metabolites increased to cope with the stress. DMSO is commonly considered a co-solvent with lower toxicity. For example, in an acute oral toxicity test on mice, its median lethal dose was higher than that of ethanol (Brown et al. 1963). Although DMSO has some inhibitory effect, its good penetrability can help ferulic acid through the cell membrane, thereby increasing the bioavailability of ferulic acid.

In terms of cell morphology, the length of *E. gracilis* cells changed periodically with the light–dark cycle and photosynthesis, which was analogous to a biological clock life pattern (Lonergan 1983). At hour 6 of the light cycle, when photosynthetic capacity was at its highest, cells were highly active and they elongated maximally to capture the light. They then became shorter as photosynthetic activity decreased, which is in agreement with the results that we obtained. Thus, there is a close relationship between cell length and cell activity. As for the decrease in cell length in the negative control group and the 10 mg/L ferulic acid treatment group, we inferred that the co-solvent DMSO might produce a large number of free radicals and ROS that have some negative effects on cell physiology and photosynthesis, resulting in a shorter cell length. In addition, *E. gracilis* cells would alter the production of certain metabolites under stress conditions. Because these lack a rigid cellulose wall and their pellicle is flexible, the cell shape would become round, resulting in a decrease in the median cell length. Compared with the negative control group, cells of the ferulic acid-treated groups recovered their normal morphology. Because of its antioxidant property, ferulic acid could scavenge toxic free radicals and ROS and relieve the stress induced by DMSO, improving the external environment for *E. gracilis* cells and restoring cells to an active state.

The decrement in total chlorophyll (which here mainly refers to chlorophyll a and chlorophyll b) was observed in the negative control group, but with the addition of ferulic acid, the proportion of total chlorophyll was recovered, especially chlorophyll a. The content of total chlorophyll reflects the plant growth state in a certain sense. Under adversities, such as drought, salinity, low temperature, pollution, or element deficiency, the content and composition of chlorophyll will be severely affected as the structure of chlorophyll is unstable (Hörtensteiner and Kräutler 2011). The serious decrease in chlorophyll content in the presence of DMSO is further proof of our previous hypothesis that the DMSO will negatively affect microalgal growth and activity. Moreover, we found that the ratio of chlorophyll a to chlorophyll b in the negative control group was also lower than that of the control group. This might be attributed to inhibition in the synthesis of chlorophyll a under stress or the conversion of chlorophyll a into chlorophyll b (Folly and Engel 1999). With increasing ferulic acid concentrations, the chlorophyll content was greatly improved to much higher than that of the two control groups, meaning that the environmental stress and restriction of chlorophyll synthesis were alleviated and cells could capture more light for their growth. It is reasonable that ferulic acid enhances the capacity of *E. gracilis* cells for utilizing the light source, resulting in a greater cell length and more vigorous growth.

The increase in paramylon content in the negative control group was likely due to a stress response, resulting in round cells with a shorter median cell length (Fig. 3) as well as slow motion and loss of vitality. *E. gracilis* cells under stress tended to change their metabolism to adapt to the new environment (Hayashi et al. 1994). Paramylon storage would increase when *E. gracilis* cells grew slowly under unfavorable conditions, while paramylon was consumed in groups that showed rapid growth (Rodríguez-Zavala et al. 2010). This explains why the paramylon content in ferulic acid treatment groups was lower than that in the negative control group. Light energy captured by cells and chemical energy stored as photosynthetic products were likely used for cell division in ferulic acid treatment groups, whereas in the negative control group they were mainly stored in the form of paramylon.

In conclusion, the significant growth promotion of *E. gracilis* and enhanced valuable metabolite production were achieved by simply adding ferulic acid from rice bran. It would be better to replace DMSO with a more harmless and efficient co-solvent, even though ferulic acid can alleviate the inhibitory effect of DMSO. The effect of ferulic acid on microalgae is supposed to differ with the strain, and the applicability of ferulic acid to commercially valuable strains such as *Arthrospira platensis*, *Botryococcus braunii* and *Haematococcus pluvialis* will be of great interest.

## Abbreviations

CM: Cramer–Myers
DMSO: dimethyl sulfoxide
ROS: reactive oxygen species
IAA: indoleacetic acid
